# Structural evaluation and bioethanol production by simultaneous saccharification and fermentation with biodegraded triploid poplar

**DOI:** 10.1186/1754-6834-6-42

**Published:** 2013-03-21

**Authors:** Kun Wang, Haiyan Yang, Wei Wang, Run-cang Sun

**Affiliations:** 1 Institute of Biomass Chemistry and Technology, Beijing Forestry University, Beijing, China; 2 Institute of Microbiology, Beijing Forestry University, Beijing, China; 3 State Key Laboratory of Pulp and Paper Engineering, South China University of Technology, Guangzhou, China

**Keywords:** Biodegradation, Lignocellulose, Simultaneous saccharification and fermentation, Bioethanol, *Trametes velutina*

## Abstract

**Background:**

Pretreatment is a key step to decrease the recalcitrance of lignocelluloses and then increase the digestibility of cellulose in second-generation bioethanol production. In this study, wood chips from triploid poplar were biopretreated with white rot fungus *Trametes velutina* D10149. The effects of incubation duration on delignification efficiency and structural modification of cellulose were comparably studied, as well as the digestibility of cellulose by simultaneous saccharification and fermentation (SSF).

**Results:**

Although microbial pretreatments did not significantly introduce lignin degradation, the data from SSF exhibited higher cellulose conversion (21-75% for biopretreated samples for 4–16 weeks) as compared to the untreated poplar (18%). In spite of the essential maintain of crystallinity, the modification of lignin structure during fungal treatment undoubtedly played a key role in improving cellulose bioconversion rates. Finally, the ethanol concentration of 5.16 g/L was detected in the fermentation broth from the cellulosic sample biodegraded for 16 weeks after 24 h SSF, achieving 34.8% cellulose utilization in poplar.

**Conclusion:**

The potential fungal pretreatment with *Trametes velutina* D10149 was firstly explored in this study. It is found that the biopretreatment process had a significant effect on the digestibility of substrate probably due to the removal and unit variation of lignin, since the crystallinities of substrates were rarely changed. Additional investigation is still required especially to improve the selectivity for lignin degradation and optimize the digestibility of cellulose.

## Background

Lignocellulose is the major component of biomass, consisting of three types of polymers, cellulose, hemicelluloses and lignin that are strongly intermeshed and chemically bonded by non-covalent forces and by covalent cross-linkages [1]. Many scientific challenges remain in understanding the recalcitrancy of lignocelluloses, and numerous chemical and physical methods have been attempted to unlock lignin polymers from the cell wall complex [2-6]. However, these pretreatment processes are often limited by the lack of selectivity to the target component, together with high energy requirement, economical feasibility and environmental unfriendliness. Thus, effective, low-cost, and green biopretreatment under mild conditions and low energy consumption has manifested the superiority over the aforementioned chemical means [7].

Nature has created a mixture of enzymatic complexes, which are capable of opening the complex structure of lignin molecules by selectively cleaving the chemical bonds between the lignin units without using/releasing any environmentally harsh chemicals [8]. Thereby, it is considered to be an environmentally friendly process with its own advantages, including no chemicals, no required special reactor, no waste and no inhibitor to fermentation. In fact, biological pretreatment has long been studied in the pulping process to save energy, increase pulp quality and reduce environmental impacts [9]. White-rot fungi, as the most promising microorganisms for selective lignin degradation, have been receiving extensive attention for biodelignification of lignocellulosic biomass. It has the ability to produce lignolytic enzymes, including manganese peroxidases (MnPs), lignin peroxidases (LiPs) and esterases (ESTRs) [10], as well as H_2_O_2_ generating enzyme systems (copper radical oxidases, aryl alcohol oxidases, glyoxal oxidases etc.), which play key roles during the lignin degradation/modification process [11,12]. Nowadays, several basidiomycetes such as *Phanerochaete chrysosporium*, *Ceriporiopsis subvermispora*, *Phlebia subserialis*, and *Pleurotus ostreatus* have been examined on different lignocellulosic substrate to evaluate their delignification efficiency. As known, most white-rot fungi simultaneously degrade carbohydrates (cellulose and hemicelluloses) and lignin, resulting in the homogeneous decay of the cell wall. Meanwhile, some species preferentially degrade lignin and part of hemicelluloses, dissolving the middle lamella and then creating the defibrillation effect. Due to the deconstruction of the impact matrix in biomass, the accessibility of cellulose for cellulase is improved and the production of sugars during bioconversion process is significantly enhanced [13-15]. Baba et al. reported that biopretreatment with white-rot fungi resulted in significantly higher sugar yield (>35%) than untreated softwood (10.2%) [16]. It was also reported that the enzymatic digestibilities of corn stover, which had been pretreated with *Cyathus stercoreus* and *P. chrysosporium*, were 3.75 and 1.26 times greater than that of untreated sample, respectively [17]. Different white rot fungus varies greatly in the relative rates, where the degradation of lignin and carbohydrates occur in lignocelluloses. Zhang et al. screened 34 kinds of white rot fungi and found only two isolates were suitable for the biopretreatment process [18]. Recently, a new fungus, *Trametes velutina* D10149, was isolated and identified in Institute of Microbiology, Beijing Forestry University. Thereby, it is necessary to investigate the biodegradation pattern and evaluate the pretreatment efficiency on bio-ethanol production by *Trametes velutina* D10149, in order to fully exploit the potential of this fungus.

In the present study, biological delignification of triploid poplar (*Populus tomentosa* Carr.) using white-rot fungus *T. velutina* D10149 was attempted. The methods of wet chemistry, X-ray diffraction (XRD), Fourier transform infrared (FTIR), and scanning electron microscopy (SEM) were applied to characterize and gain insights on the delignification process and structural modification of cellulose in lignocelluloses. The biological pretreatment was further evaluated by ethanol yield from bioconversion process, simultaneous saccharification and fermentation (SSF), which will better meet the requirement for 2^nd^ generation bio-ethanol production.

## Results and discussion

### Delignification process evaluation

Lignification is considered to be a primary factor limiting the biodegradation of the cell wall by rumen microbes, and the knowledge of lignin content in the plant is consequently of primary importance to access the mechanisms involved in the inhibition of structural carbohydrate digestion. There are several analytical procedures for determining the lignin content, in which acetyl bromide soluble lignin (ABSL) method was considered to be a fast/convenient method and useful to predict in vitro digestibility [19]. As expected, the values of ABSL content were gradually decreased from 33.4% (control) to 24.6% as prolonging the incubation duration (Table 1). The maximum degree of the delignification was determined to be 61% (taking into account 46% weight loss of biodegraded sample for 16 weeks) in this work, which is in the same order of magnitude as pointed out by Zhang et al. [20]. Slight decrease of lignin was observed in the first 4 weeks incubation, and similar behavior has been found by Dinis et al. [21]. They detected the ligninolytic system of four white rot fungi, and noted that the maximums of manganese-dependent peroxidase (MnP), lignin peroxidase (LiP) and laccase activities varied greatly at different fermentation times. Since lignin degradation depended on the synergistic role among the ligninolytic enzymes, it usually occurred later and gradually achieved the maximum degradation rate after 30 days [20].

**Table 1:** The content of lignin (ABSL, wt%), infrared ratios and crystallinity indices of untreated and biopretreated cellulosic samples after different incubation time

**Sample**^**a**^	**ABSL**^**b**^	**FT-IR**		***CrI***^**c**^	***CrI***^**d**^
		**LOI (α**_**1437 **_**cm**^**-1**^**/α**_**899 **_**cm**^**-1**^**)**	**TCI (α**_**1378 **_**cm**^**-1**^**/α**_**2900 **_**cm**^**-1**^**)**		
*C*_0_	33.4	0.92	1.01	40.7	38.2
*C*_4_	32.5	0.92	1.04	41.7	42.8
*C*_8_	26.6	0.89	0.95	41.3	43.2
*C*_12_	25.1	0.93	1.14	40.4	42.1
*C*_16_	24.6	0.85	1.03	40.6	42.2

^a^*C*_x_ represents the cellulosic residues, while the subscript X (4, 8, 12, and 16) represents the different incubation weeks. *C*_0_ was obtained without the biodegradation treatment as a control; ^b^ABSL is abbreviation of acetyl bromide soluble lignin, and the standard deviations are less than 5%; ^c^*CrI* was calculated based on the height of the peak corresponding to (002) lattice plane (*I*_002_) and the minimum between 110 and 002 lattice planes (*I*_am_) as follow: $C_{r}I\left(\%\right)=\frac{I_{002}-I_{\mathrm{am}}}{I_{\mathrm{am}}}\times 100$, and the standard deviations are less than 2%; ^d^*CrI* was calculated from the ratio of the crystalline area over the total area, where separation of crystalline (86–92 ppm) and amorphous (79–86 ppm) fractions were based on Guassian line shape function, and the standard deviations are less than 2%.

**Figure 1:**
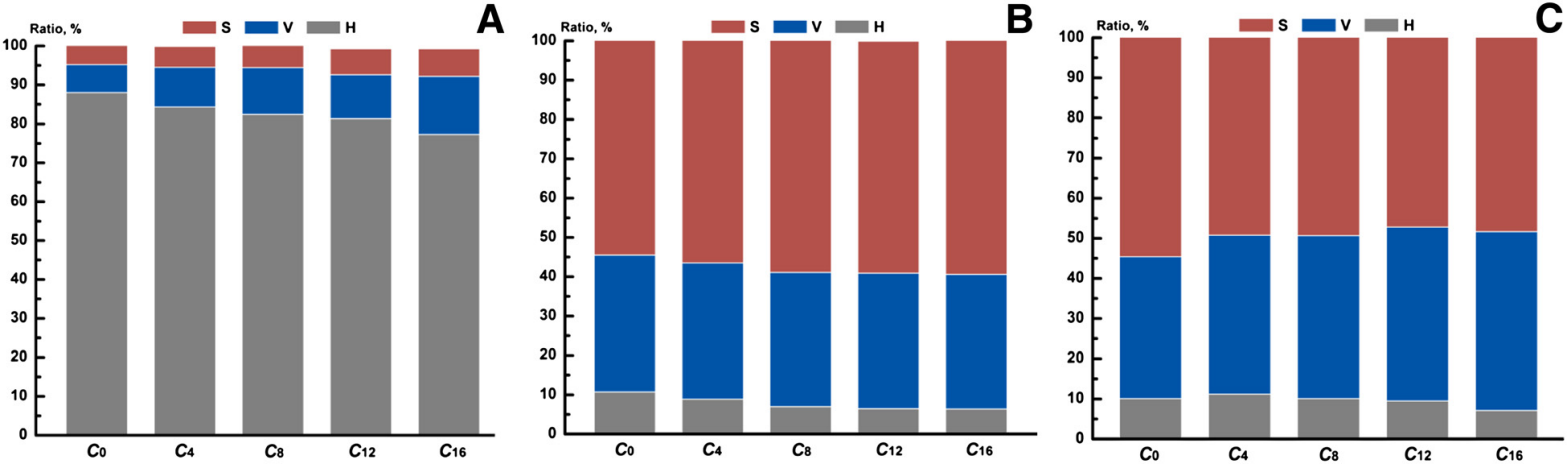
**Unit ratio of the associated lignin in the untreated and biopretreated cellulosic samples (the subscript X represents the different incubation weeks). A** 1N NaOH (ester-bound phenolics); **B** 4N NaOH (ether-bound phenolics); **C** alkaline nitrobenzene oxidation (non-condensed phenolics). (S represents the total mass of syringaldehyde, syringic acid and acetosyringone; V represents the total mass of vanillin, vanillic acid and acetovanillone; H represents the total mass of *p*-hydroxybenzoic acid and *p*-hydroxybenzaldehyde).

**Figure 2:**
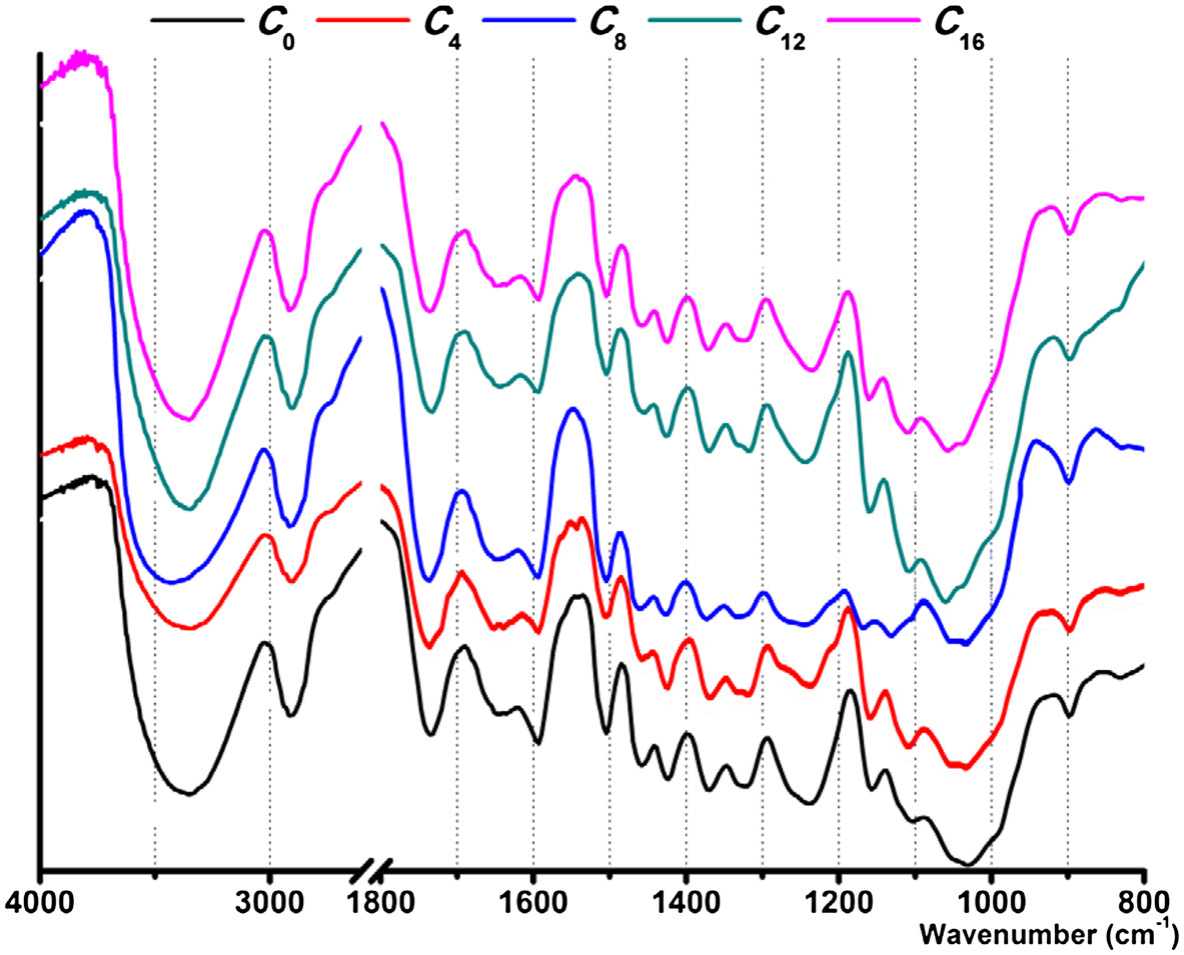
FTIR spectra of the untreated and biopretreated cellulosic samples (the subscript X represents the different incubation weeks).

The ratios of phenolic composition in poplar cell wall are presented in Figure 1. Hydroxycinnamic acids, as bifunctional molecules with carboxylic and phenolic bonding sites, are usually involved in bridges through ester/ether linkages between lignin fragments and distinguished by treatment with alkali solution at different temperature and concentration. Obviously, the H unit predominated in ester bound cell well phenolics in all samples (Figure 1A), arranging from 88.0% to 77.3%. These data are in accordance with the conclusion that the ester linkages in aspen wood lignin are mainly *p*-hydroxybenzoate groups [22]. As the incubation processed, the yields of the detectable phenolics were gradually decreased (Table 2), as well as the H unit proportion (Figure 1A). Although the biodegradation data for poplar were seldom involved, the release of hydroxy-cinnamic acids in several agroindustrial byproducts could be used as references. Dinis et al. reported that the decrease of esterified hydroxycinnamic acids underwent two stages: the rapid stage, above 70% decrease occurred at 21 days of incubation; the stable stage, the content of esterified hydroxycinnamic acids remained fairly stable for the period between 21 and 28 days incubation [21]. Although longer incubation time was employed in current study, similar trend was also observed. It indicated that all the necessary enzymes were probably produced to act on the target linkages and release hydroxycinnamic acids. In terms of ether bonds, the majority of the cell well phenolics was S-derived lignin unit (54.5%-59.4%) (Figure 1B), and the yields of monolignol derivatives were expectably decreased as incubation processed (Table 3). Despite the S/G ratio almost maintained around 1.56 to 1.73, the relative proportion of H unit was slightly reduced from 10.7% to 6.4%. It could be speculated that the ligninolytic system produced by *Trametes velutina* D10149 non-selectively cleaved the ether bonds between lignin substructures, relatively preferring to the H-derived lignin unit. The composition of the phenolic compounds was obtained from alkaline nitrobenzene oxidation, reflecting the structural features of the side chain and extent of carbon−carbon linkages presented in the lignin. As expect, the major products were identified to be syringaldehyde and vanillin in the sample C_0_, representing 49.4% and 31.9% of the phenolic compounds, respectively (Table 4). As the biodegradation of lignin component, the detectable phenolic acids and aldehydes were gradually reduced from 48.99 to 35.27 μg/mg (weight % of cellulosic samples). Clearly, the structural alteration of lignin in white rot fungi biodegradation primarily involved a preferential degradation of syringyl units, evidenced by the decreased S/G ratio from 1.55 (C_0_) to 1.24 (C_4_), to 1.21 (C_8_), to 1.09 (C_12_), and to 1.08 (C_16_) (Figure 1C). Similar phenomena were observed on poplar biodegradation by Ke et al. [23]. Based on the analysis of many methods (CP/MAS, ATR-FTIR, Py-GC/MS, etc.), they concluded that the preferential lignin degradation site was at the S-units. Tai et al. isolated the ether-insoluble fraction from birch wood decayed by the white rot fungus *Phanerochaete chrysosporium*, and also found that syringyl type substructures were more susceptible to oxidative cleavage of C_α_–C_β_ bond than guaiacyl type substructure [24]. On the basis of the component identification, the lignin-peroxidase-initiated, one-electron-transferring enzyme-oxidation homolytically cleaved C_α_–C_β_ bond to produce 4-*O*-alkylated vanillin/syringaldehyde, and then generated the corresponding vanillic/syringic acids through radical-intermediate-induced autoxidations [25]. However, the structural variation of lignin was too insignificant to be clearly reflected in the FTIR spectra (Figure 2), as well as its low signal intensity on the lignocellulosic material.

**Table 2:** **Yields of monolignol derivatives (w/w, μg/mg)**^**a **^**of untreated and biopretreated cellulosic samples, obtained from 1N NaOH extraction at room temperature**

**Samples**^**b**^	***C***_**0**_	***C***_**4**_	***C***_**8**_	***C***_**12**_	***C***_**16**_
*p*-hydroxybenzoic acid	1.81	1.40	1.30	1.22	1.09
*p*-hydroxybenzaldehyde	0.03	ND^c^	0.01	ND	ND
vanillic acid	0.08	0.10	0.11	0.10	0.13
syringic acid	ND	ND	ND	ND	ND
vanillin	0.07	0.07	0.08	0.07	0.08
syringaldehyde	0.11	0.09	0.09	0.10	0.10
Total	2.09	1.66	1.59	1.50	1.41

^a^The standard deviations are less than 5%; ^b^Corresponding to the cellulosic residues in Table 1; ^c^Not detectable.

**Table 3:** **Yields of monolignol derivatives (w/w, μg/mg)**^**a **^**of untreated and biopretreated cellulosic samples, obtained from 4N NaOH extraction at 170°C**

**Samples**^**b**^	***C***_**0**_	***C***_**4**_	***C***_**8**_	***C***_**12**_	***C***_**16**_
*p*-hydroxybenzoic acid	3.29	2.62	2.12	1.77	1.68
*p*-hydroxybenzaldehyde	0.27	0.09	0.05	0.07	0.09
vanillic acid	1.32	1.30	1.14	1.05	1.06
syringic acid	1.27	1.27	1.02	1.03	1.08
vanillin	9.47	8.38	8.41	8.04	7.94
syringaldehyde	15.01	14.30	16.22	14.58	14.37
acetovanilline	0.76	0.82	1.02	0.62	0.51
acetosyringone	1.80	1.59	0.99	1.04	1.06
Total	33.20	30.38	30.98	28.18	27.79

^a^The standard deviations are less than 5%; ^b^Corresponding to the cellulosic residues in Table 1.

**Table 4:** **Yields of phenolic acids and aldehydes (w/w, μg/mg)**^**a **^**from alkaline nitrobenzene oxidation of untreated and biopretreated cellulosic samples**

**Samples**^**b**^	***C***_**0**_	***C***_**4**_	***C***_**8**_	***C***_**12**_	***C***_**16**_
*p*-hydroxybenzoic acid	4.77	4.70	3.95	3.40	2.34
*p*-hydroxybenzaldehyde	0.20	0.15	0.07	0.05	0.16
vanillic acid	1.11	1.32	0.78	1.01	1.57
syringic acid	2.22	2.30	1.72	1.69	2.20
vanillin	15.63	15.90	15.40	14.40	14.18
syringaldehyde	24.18	19.13	17.93	15.12	14.83
acetovanilline	0.54	ND^c^	ND	0.25	ND
acetosyringone	0.34	ND	ND	0.26	ND
Total	48.99	43.49	39.86	36.18	35.27

^a^The standard deviations are less than 5%; ^b^Corresponding to the cellulosic residues in Table 1; ^c^Not detectable.

### Crytallinity and morphologic analysis

**Figure 3:**
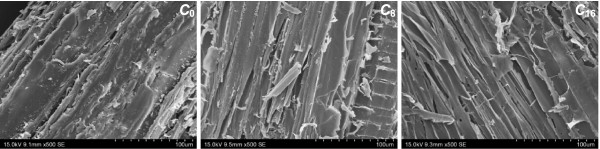
**SEM micrographs (× 500).** Control (C_0_>) and biopretreated poplar samples (C_8_ for 8 weeks incubation and C_16_ for 16 weeks incubation) with *T. velutina* D10149.

Contrary to the conventional chemical analysis, FTIR spectroscopy has been used as a rapid technique for obtaining crystal information of cellulosic samples with advantages of small sample requirement, short analysis time, and no destruction. Four peaks closely related to the crystal system and degree of intermolecular regularity were concerned, and two infrared ratios, α_1437_ cm^-1^/α_899_ cm^-1^ (lateral order index (LOI)) [26] and α_1378_ cm^-1^/α_2900_ cm^-1^ (total crystallinity index (TCI)) [27], were calculated and listed in Table 1, respectively. Hardly any difference was observed in all cellulosic samples, even when the incubation time had been prolonged to 16 weeks. The biodegradation of crystalline cellulose generally involves the action of both endo- and exo-acting cellulases. When the brown-rot fungus (*Fomitopsis palustris*) was used to pretreat *Cryptomeria japonica* trees, the generated enzymes complex resulted in the obvious decrease of crystallinity from 48.9% to 9.1% after 12 weeks of incubation [28]. However, no data on cellulose crystallinity decrease by white-rot fungi was reported until now, indicating that the ligninolytic system of white rot fungi had a negligible effect on the crystalline cellulose. The *CrI* values of all samples were also calculated from the corresponding XRD (Figure 3) and solid-state cross polarization/magic angle spinning nuclear magnetic resonance (CP/MAS NMR) patterns (data not shown), and the data were listed in Table 1. As the data shown, it is in agreement with the conclusion from the FTIR analysis, and further confirmed that the crystallinity almost remained essentially the same in the biodegradation process.

**Figure 4:**
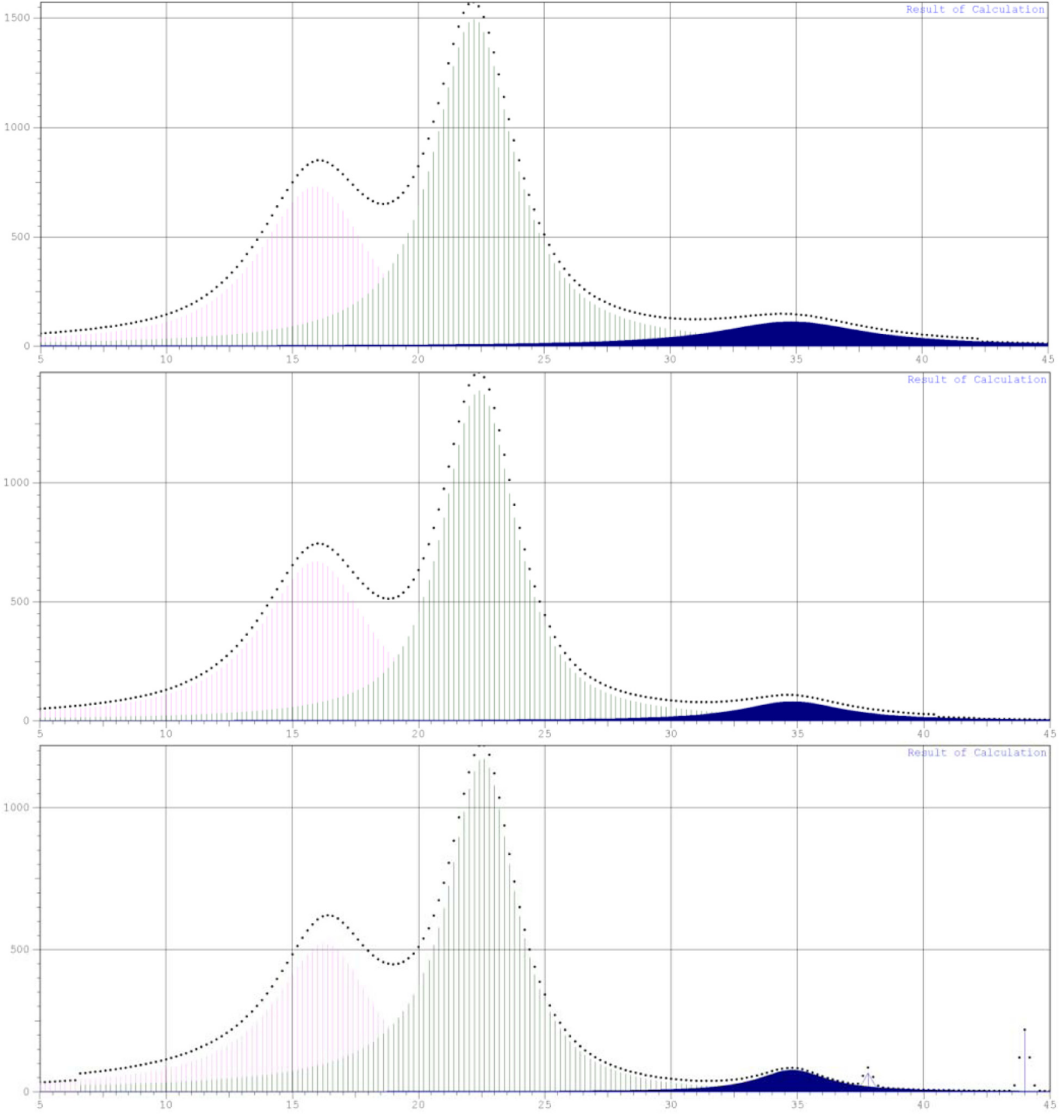
**X-ray diffractograms.** Untreated (C_0_) and fungal-pretreated (C_4_ for 4 weeks incubation and C_16_ for 16 weeks incubation) poplar.

The surface area is generally considered as important role in the accessibility and adsorption of enzymes. Clearly, the SEM images demonstrated that the biological pretreatment with white rot fungus *Trametes velutina* D10149 led to the evident damage on the intact cell structure (Figure 4), which will benefit for enhancing digestibility of pretreated poplar with SSF. The native material (C_0_) displayed a regular and compact surface structure, the bast fibers arranged in bundles. After 8 weeks incubation, the apparent changes on surface were observed (C_8_), including the presence of cracks and ridges, the exposure of internal cell wall, and the disappearance of cohesion within the fibers. However, the penetration of cellulose fibers by hyphal was not obvious from SEM. Prolonging incubation period to 16 weeks (C_16_), the cell wall was further attached by the fungus or the extracellular enzymes secreted by the fungus, presenting more cracks and scars. Fibril separation was also observed since the degraded fibers were split in the direction of the fiber axis. Overall, it could be speculated that the defibrillation and delignification co-effect of biodegradation released a large reactive area on the fiber surface, consequently improving the accessibility of cellulose and the bioconversion efficiency.

### Products in SSF

**Figure 5:**
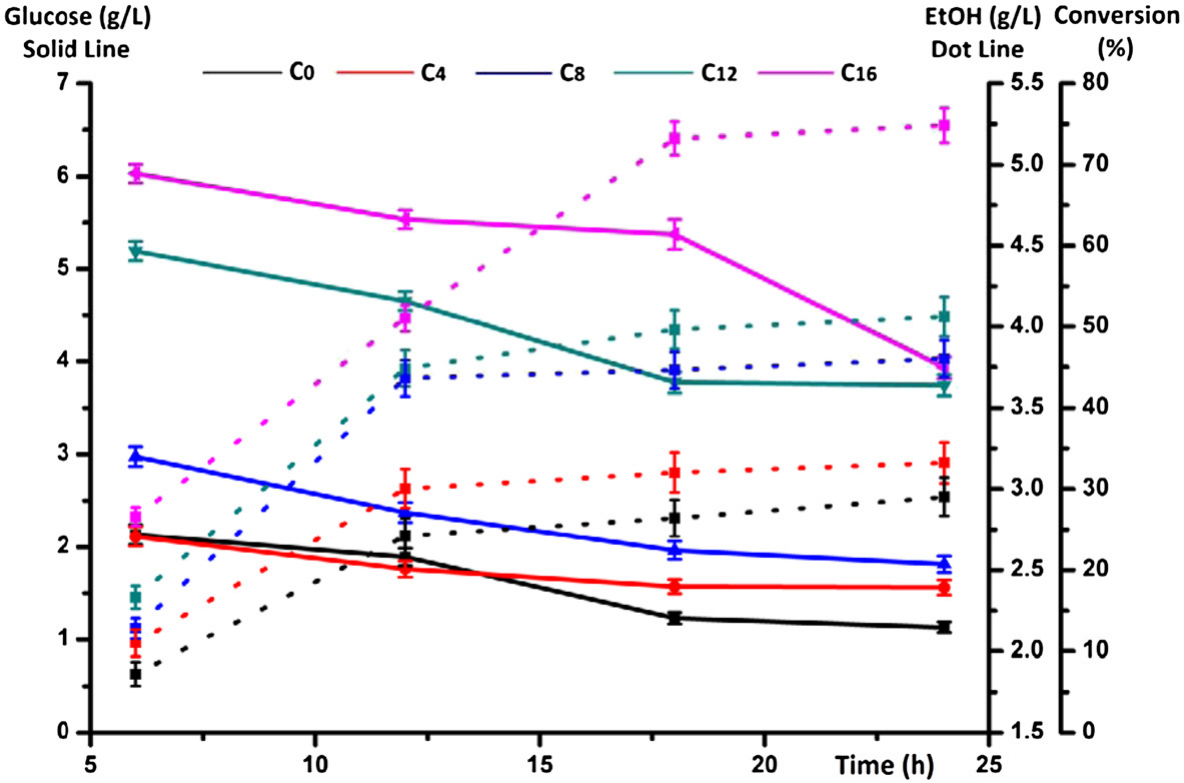
**Time courses of glucose, ethanol yield (g/L) and cellulose conversion (%) during the simultaneous saccharification and fermentation (SSF) of untreated and biodegradation-treated poplar with white rot fungi *Trametes velutina *D10149.**

SSF was conducted to evaluate ethanol production from fungal pretreated poplar. In SSF, enzymatic hydrolysis and fermentation are run simultaneously, which has been shown to have several advantages over a separate process (separate hydrolysis and fermentation, SHF), including lower enzyme inhibition, less processing time, and higher ethanol yield [29]. Since enzymatic hydrolysis is the limiting factor in SSF at the low yeast concentration [30], the concentrations of glucose and ethanol versus time were measured and shown in Figure 5. After 24 h of SSF, the highest ethanol yield (75%) was reached from the cellulosic sample biodegraded for 16 weeks (C_16_), which is equivalent to the ethanol concentration of 5.16 g/L in the fermentation broth. From the raw poplar (C_0_), the final concentration of ethanol was only 2.95 g/L, corresponding to less than 30% cellulose conversion. Clearly, prolonging incubation duration could gradually increase the cellulose conversion. This phenomenon was closely related to the partial removal of lignin and the modification of lignin macromolecules during the biodegradation treatment. Although the adsorption of cellulase on lignin significantly affects the enzymatic hydrolysis of cellulose, only 43% lignin removal is needed to render the biomass digestible [31]. Based on the ABSL results above, the relative content of lignin in the cellulosic samples just decreased from 33.4% (C_0_) to 24.6% (C_16_). Thereby, the modification of lignin structure during fungal treatment undoubtedly played a key role in improving cellulose bioconversion rates. It could be speculated that the cleavage of ester and ether bonds probably decreased the hydrophobicity of lignin macromolecules and the unproductive binding of cellulase, correspondingly. Moilanen et al. treated the steam pretreated spruce with laccase and found that a lower amount of cellulases were adsorbed to lignin than the untreated sample [32]. Meanwhile, the partially blocking effects of lignin on the accessible pores and surfaces of cellulose were probably reduced by lignin removal with biodegradation. In addition, the decreasing trends of glucose concentration were similar, remaining less than 4.5 g/L in the final broth. Combining with the weight losses [33], biodegradation with white-rot fungus *Trametes velutina* D10149 for 16 weeks was proved to be the best choice, achieving 34.8% cellulose utilization in poplar. Best conversion result obtained was 75% of the theoretical value, indicating that the process (biodegradation, delignification and/or saccharification/fermentation) should be optimized to achieve higher conversion rate in our future work.

## Conclusions

Selective white rot fungus has shown potential for lignocelluloses pretreatment. In this study, a new fungal isolate, *Trametes velutina* D10149, was used in the biological pretreatment to enhance the digestibility of triploid poplar. Although no unique and significant selectivity for lignin degradation was observed in biodegradation process, the ethanol production was achieved 5.16 g/L in the fermentation broth after 24 h SSF from fungal-pretreated poplar substrate. Based on wet-chemical analysis of remaining lignin, the structural variation in lignin macromolecules was important for improving lignocelluloses bioconversion efficiency.

## Methods

### Raw materials and chemicals

Triploid poplar (*Populus tomentosa* Carr.) of 3-year-old was cut from the suburb of Beijing, China. After being processed through a combination of chipping and milling, the fraction passing 40-mesh was collected and used throughout the study. The main components of the wood were determined as: glucose 44.4±0.6%, xylose 23.0±0.5%, Klason lignin 19.4±0.3%, and acid soluble lignin 4.9±0.1% (weight % of starting material). All chemicals are of analytical grade unless otherwise mentioned.

### Fungal strains and biopretreatment process

The white rot fungus *Trametes velutina* D10149 was isolated from Jilin province in China, and preserved on 2% (w/v) malt-extract agar (MEA) plates at 4°C in Institute of Microbiology, Beijing Forestry University. A plug of the fungi activated in 100 mL basic medium (containing glucose 20 g/L, yeast extract 5 g/L, KH_2_PO_4_ 1 g/L, MgSO_4_ 0.5 g/L, and VB_1_ 0.01 g/L). After been cultured on a rotary shaker at 28°C with a speed of 150 rpm, mycelial pellets were harvested after 5 d and mixed with 100 ml distilled water. This suspension would act as inoculums.

The biological pretreatment with *T. velutina* D10149 was carried out in a 250-mL Erlenmeyer flask with 5 g of air-dried poplar and 12.5 mL of distilled water. The samples were sterilized in the autoclave for 20 min at 121°C and inoculated with 5 mL inoculums. The culture was incubated statically in 28°C for 4–16 week. The non-inoculated sample was served as the control. All experiments were performed in triplicate.

### Enzyme and yeast

The Cellulast 1.5L (cellulase) and Novozyme 188 (*β*-glucosidase) were kindly provided by Novozymes Investment Co. Ltd. (Beijing, China). The microorganism used for fermentation was *Saccharomyces cerevisiae* in the form of dry yeast (thermal resistant) (Angel Yeast Company Ltd, Yichang, China). Dry yeast was activated in 2% glucose solution at 40°C for 20 minutes, then at 34°C for 2 hour.

### Evaluation of delignification and monolignol

The associated lignin was analyzed through spectrophotometric method, which is easy and rapid for determining the total lignin concentration of a cell wall sample [19]. The procedure was described in detail in a previous paper [34], and the concentration of ABSL was calculated by adopting the appropriated absorption coefficient, 18.21 mL mg^-1^ cm^-1^[35]. The ester-bound, ether-bound and non-condensed phenolics were analyzed with 1N NaOH at room temperature, 4N NaOH at 170°C and alkaline nitrobenzene oxidation processes, respectively. The separation and identification of phenolics was achieved with a HPLC system (1200 series, Agilent Technologies, USA) on a ZORBAX Eclipse XDB-C18 column (4.6 × 250 mm) by comparison of retention times and UV spectra (DAD, diode array detector) of the eluting peaks and the authentic standard compounds (Sigma–Aldrich Corp.; St. Louis, MO, USA) [36].

### Structural characterization

FTIR spectra of the cellulosic samples were recorded from an FTIR spectrophotometer (Tensor 27, Bruker, Germany) in the range 4000 to 800 cm^-1^, using a KBr disc containing 1% finely ground samples. Shimadzu XRD-6000 instrument (Japan) was used to examine the crystallinity, scanning from 5° to 35° 2θ by a goniometer at a scanning speed of 5°/min. The relative crystallinity is commonly measured as a ratio between the diffraction portion from the crystalline part of the sample and the total diffraction from the same sample. The surface characteristics of fungal treated substrates were observed by SEM, which was conducted with an S-3400N instrument (HITACHI, Japan) at acceleration voltages of 10 kV. Samples were firstly coated with gold-palladium in a sputter coater (E-1010, HITACHI, Japan).

### Simultaneous saccharification and fermentation

The SSF experiments were performed under non-sterile conditions in 50 mL Erlenmeyer flasks sealed with a rubber stopper fitted with a one-way air valve to maintain an anaerobic environment. Each fermentation flask was composed of 0.5 g of untreated and fungal-treated substrates and 9 mL of nutrient medium, containing 10 g/L yeast extract and 20 g/L peptone in 50 mM sodium acetate buffer (pH 4.8). The insoluble substrates and the fermentation medium were sterilized at 121°C for 20 min. Then, 30 FPU/g substrate of cellulase (Celluclast 1.5L), 60 IU/g substrate of *β*-glucosidase (Novozyme 188) and 3 g/L yeast were added. Fermentation was carried out at 40°C for 24 h with shaking at 120 rpm. Aliquots of 0.5 mL were withdrawn and centrifuged at 10000 rpm for 5 min, and the supernatants were subjected to fermentation products analysis. All fermentations were performed in triplicate [37].

### Fermentation analysis

Each sample was filtered through a 0.22 μm filter and diluted appropriately with deionized water. Quantitative analysis for ethanol, glucose and xylose was performed on above HPLC system (1200 series, Agilent Technologies, USA) equipped with a refractive index detector. The separation was achieved using an Aminex HPX-42H column (300×7.8 mm i.d.; Bio-Rad Labs, Richmond, CA, USA) at 65°C with 4 mM H_2_SO_4_ as eluent at a flow rate of 0.6 mL/min. The cellulose conversion was calculated using the following formula [38]: $\mathrm{\%Cellulose}\;\mathrm{Conversion}=\frac{{\left[\mathrm{EtOH}\right]}_{f-}\left[\mathrm{EtOH}\right]}{0.51\left(f\left[\mathrm{Biosmass}\right]\times 1.111\right)}\times 100\%$1.

## Competing interests

The authors declare that they have no competing interests.

## Authors’ contributions

KW and RS designed and coordinated the study, WW and HY carried out the experiments, and KW and HY analyzed the results. KW and HY wrote the paper, and RS reviewed the paper. All authors read and approved the final manuscript.
